# Hydroxyethyl starch - the importance of being earnest

**DOI:** 10.1186/1757-7241-21-61

**Published:** 2013-08-09

**Authors:** Daniel Chappell, Matthias Jacob

**Affiliations:** 1 Department of Anaesthesiology, University Hospital of Munich, Nussbaumstrasse 20, 80336 Munich, Germany

**Keywords:** Colloids, Crystalloids, Fluid therapy, Hydroxyethyl starch, Sepsis

## Abstract

Despite ongoing controversial expert discussions the European Medicines Agency (EMA) recently recommended to suspend marketing authorisations for hydroxyethyl starch. This comment critically evaluates the line of arguments. Basically, the only indication for a colloid is intravascular hypovolemia. Crystalloid use appears reasonable to compensate ongoing extracellular losses beyond. In the hemodynamically instable patient this leads to the distinction between an initial *resuscitation phase* where colloids might be indicated and a crystalloidal *maintenance phase* thereafter. It is important to bear this in mind when reevaluating the studies the EMA referred to in the context of its recent decision: i) VISEP compared ringer’s lactate to 10% HES 200/0.5 in septic patients and found an increased incidence of renal failure in HES receivers. Unfortunately, study treatment was started only after initial stabilization with HES, randomizing hemodynamically stable patients into a rational (crystalloids) and an irrational (high dose starch until ICU discharge) maintenance treatment. ii) 6S compared ringer’s acetate to 6% HES 130/0.42 for fluid resuscitation in septic patients and found an increased need of renal replacement therapy and a higher mortality in the HES group. However, patients of both groups were again randomized only after initial stabilization with colloids, the actual comparison was, therefore, again rational vs. irrational. Beyond that, the documentation is partly fragmentary, leaving many important questions around the fate of the patients unanswered. iii) CHEST randomized ICU patients to receive saline or 6% HES 130/0.4 for fluid resuscitation. Actually, despite partly discussed in a different way, this trial showed no relevant differences in outcome.

In all, two studies showed what happens to septic patients if starches are used in a way we do not observe in daily practice. The third one actually proves their safety. The benefit of perioperative goal-directed preload optimization using starches is unquestioned. Taking these informations into account, the recommendation of the EMA starches to be generally dangerous remains mysterious and incomprehensible. An authority being able to dictate behavior should stand clear from oppressively ending a worldwide expert discussion and step back into the role of the observer until science achieves an agreement.

## 

After years of throwing crystalloids and colloids into one pot, the last decade increasingly established a differentiation between crystalloidal substitution of extracellular losses and stabilization of cardiac preload beyond [1]. A “goal-directed” approach to the latter with colloids has been shown – at least for the perioperative setting - to reduce morbidity [2] and is implemented in the British Consensus Guidelines on Intravenous Fluid Therapy for Adult Surgical Patients (GIFTASUP) [3]. Recent evidence suggested, however, that long-term use of hydroxyethyl starches (HES) in high cumulative doses could be a problem in septic patients. The discussion even among experts is ongoing and remains controversial [4,5].

Nevertheless, on June 14th 2013 the risk assessment committee of the European Medicines Agency (EMA) recommended to suspend marketing authorisations for HES for all indications. In the following we will try to clarify whether this was scientifically justified.

## The physiological view: compartments and volume effects

Normally, two-thirds of the total body water are intracellularly, the remaining part is located extracellularly, distributing to 80% interstitially and to 20% intravascularly. These two extracellular sub-compartments are separated by the vascular barrier, sufficiently retaining macromolecules, but being freely permeable to water and electrolytes. This explains the observation that during resuscitation of a bleeding patient with isotonic crystalloids the infused amount is distributed evenly over the entire extracellular space, i.e., to 20% intravascular and to 80% interstitial [6]. Iso-oncotically prepared colloids, by contrast, remain nearly completely within a primarily hypovolemic vascular system after i.v. infusion [7]. The repeatedly expressed clinical suspicion that crystalloids used for volume resuscitation might have a similar intravascular persistence as colloids is wrong. The possible advantage of colloids over crystalloids for stabilizing cardiac preload is obvious: Crystalloids require the 4-5-fold amount to sufficiently stabilize macrohemodynamics. Most of this fluid would shift into tissue causing substantial interstitial edema [1], itself being associated with increased mortality [8]. However, colloidal volume effects of 80-100% only account for their use to correct *intravascular hypovolemia*. Isooncotic fluids infused as a *hypervolemic bolus* into the circulation of previously normovolemic patients have been shown to have a reduced volume effect of around 40%, thus, 60% being shifted towards the interstitium. The reason is a hypervolemia-related impairment of vascular barrier functioning [9].

The only rational indication for an i.v. colloid is acute intravascular hypovolemia. Therefore, colloids are not indicated in normovolemic patients. Beyond that, HES is contraindicated in patients with acute renal failure.

## The outcome-based view: evidence in fluid therapy

In 2001 the group around Emanuel Rivers [10] taught us one thing above all: failure to early hemodynamically stabilize patients in acute shock is extremely difficult to compensate for later. Quite obviously we must distinguish between an initial (6-hour) *resuscitation phase* of hemodynamically instable patients and a *maintenance phase* thereafter. During *resuscitation* volume therapy is an important part of an outcome-relevant causal therapy, deciding between life and death. *Maintenance* with fluids is only one measure within a multifactorial supportive concert and defining reliable outcome parameters is as difficult as defining a reproducible standard.

It is important to keep this in mind when evaluating the three trials the risk assessment committee of the EMA was primarily referring to when recommending against HES in general [11-13].

## VISEP, 6S and CHEST: what did they actually do?

The investigators of the VISEP trial [11] compared the use of ringer’s lactate to that of 10% HES 200/0.5 for volume replacement therapy in 537 septic patients. Receiving colloids following their protocol led to an increased incidence of renal failure and “a trend towards higher 90-day mortality”, despite being non-significant. Unfortunately, study treatment was started only up to 24 h after diagnosis of severe sepsis. As the treating physicians were not passive in between, at this time initial hemodynamic stabilisation had already been completed in the vast majority of patients, the median values of mean arterial pressure (MAP), central venous pressure (CVP), central-venous oxygen saturation (ScvO2) and lactate at study onset having been 75 mmHg, 12 mmHg, 74% and 2.2 mmol/l, respectively. This led to randomization into i) a “crystalloid” group in which 58% had already successfully received up to 1 litre of HES for initial resuscitation (remarkably, further 33% in this group received colloids *during* the trial) and ii) a colloid group which received this outdated hyperoncotic solution over a prolonged period of time outside a proper indication and in daily and cumulative dosages beyond any recommendation. Especially the latter aspect is important, as even in this study the subgroup of patients who received this HES preparation in daily amounts within the *recommended* range showed a *lower* mortality (!) than the crystalloid group.

The 6S trial [12] compared the use of ringer’s acetate to the application of 6% HES 130/0.42 for fluid resuscitation in 800 septic patients. Similar to VISEP, the authors found an increased incidence of renal replacement therapy (RRT) after the use of HES and, beyond that, a significantly higher 90-day-mortality. However, also similar to VISEP, patients were once again only randomized up to 24 h after diagnosis of severe sepsis or septic shock into i) a "crystalloid" group in which over 60% had already received up to 1,000 ml of colloid for initial resuscitation and ii) a colloid group in which the majority of patients was already hemodynamically stabilized, the baseline median values of CVP, ScvO2 and lactate having been 10 mmHg, 75% and 2.0 mmol/l, respectively. According to the recommendations of the Surviving Sepsis Campaign these values are even better than the *targets* of fluid resuscitation [14] - so most certainly not a trigger. Therefore, in this study the colloid receivers were, once again, tested for a non-indicated drug, in comparison to the rational and well established approach with crystalloidal maintenance in stable patients. Additionally, 36% of the randomized patients had renal failure already at study onset, a clear contraindication of HES. Moreover, 216 patients in both groups (27%) discontinued trial fluid during the study and 32% of the “crystalloid” group received colloids *during* (!) the trial. Nevertheless, all were included into the 90-day follow-up. The individual cause of death is not reported and several other important values, the documentation of which would have been part of the protocol, were not indicated (e.g. hematocrit, (mean) arterial pressure or data on mechanical ventilation). The objective criteria for assessing renal failure (the RIFLE-score) are reported in the supplement and show no significant differences. This is not mentioned nor discussed manuscript. Hemodynamical parameters are reported only for the first 24 hours. Although the trial theoretically went on for 90 days, the length of hospital and ICU stay is not reported and the use of study and non-study fluids is only stated for the first 3 days. It has to be concluded that we do not know enough about what actually happened to these patients. Obviously, in this trial the majority of the “crystalloid” group received colloids during initial stabilization, i.e., with a good indication and 1/3 even during the trial. After stabilization, they were randomized into a rational (crystalloids) or an irrational (colloid) maintenance protocol, receiving HES in high amounts and over a prolonged period of time. Considering the lack of a proper indication in the majority and an absolute contraindication in a large part of the patients, negative effects are not surprising. It is simply not possible to conclude pure crystalloidal treatment to be superior to the use of colloids from a study where practically every patient also in the crystalloid group received some kind of colloid.

The CHEST trial [13] randomized 7,000 patients, at mean 11 hours after ICU admission, to receive saline or 6% HES 130/0.4 for fluid resuscitation. The authors reported a main analysis which found no differences in mortality or renal function according to the RIFLE criteria, but an increased incidence of RRT after HES infusion in the non-adjusted analyses. They tried to explain this by post-hoc tests, allegedly showing higher relative risks for the HES receivers to develop renal insufficiency states “RIFLE-R” (risk of renal failure) and “RIFLE-I” (kidney injury). This is quite simply not true, as can be easily taken from Table 1, nor is it even possible. In general, post-hoc tests evaluate where effects from a main analysis might come from - as a matter of fact they cannot contradict this main analysis. Why patients with the better renal function (according to the objective RIFLE criteria) had a slightly higher rate of RRT (subjective aspect) remains unclear (7.0% vs. 5.8%, p = 0.04). The most likely explanation is that there were no standardized triggers for this supportive measure. Therefore, first of all, not more patients *required* but *received* RRT. Interestingly, in the adjusted analysis (a standard procedure to eliminate influence by, e.g., age, gender or severity of illness) there was no difference in the incidence of RRT in this trial. That actually should have led to the conclusion that, due to an improved kidney function and no differences in RRT, CHEST shows an advantage for HES. Moreover, the protocol was violated 953-times in 634 (9.5%) patients by infusing the wrong study fluid. All these patients remained in the trial. This means that there were more patients receiving the wrong fluid than RRT, making a result being influenced by chance at least possible. Mean hemodynamic values at baseline in the HES group were MAP 74 mmHg, CVP 9.5 mmHg and lactate 2.1 mmol/l, all exceeding the recommended targets of the Surviving Sepsis Campaign. This reveals hemodynamic stability and no need for colloidal fluid resuscitation. Also in this trial 36% of the patients had acute renal failure at randomisation (an already mentioned contraindication for HES) and 508 patients in the saline group had received HES prior to randomisation. Use of other colloids was not reported. Notably, 30% of the patients were septic and in this subgroup no differences in mortality, renal failure or renal replacement therapy were observed.

**Table 1:** **A modified excerpt of the original ‘Figure S3’ in the supplementary appendix of the CHEST trial**[13]

**RIFLE category and component**	**HES n/N (%)**	**Saline n/N (%)**	**Relative risk 95% confidence interval**	**p**	**Result**
RIFLE-R (risk)	1788/3309 (54.0)	1912/3335 (57.3)	0.94 (0.90-0.98)	0.007	HES better
Creatinine increase (x1,5) from baseline	462/3149 (14.7)	415/3171 (13.1)	1.12 (0.99-1.27)	0.07	n.s.
Urine output <0.5 ml/kg/h x 6 h	1701/3230 (52.7)	1846/3266 (56.5)	0.93 (0.89-0.97)	0.002	HES better
RIFLE-I (injury)	1130/3265 (34.6)	1253/3300 (38.0)	0.91 (0.85-0.97)	0.005	HES better
Creatinine increase (x2) from baseline	245/3149 (7.8)	191/3171 (6.0)	1.29 (1.08-1.55)	0.006	Saline better
Urine output <0.5 ml/kg/h x 12 h	1077/2977 (36.2)	1200/3024 (39.7)	0.91 (0.85-0.97)	0.005	HES better

The results clearly contradict the statement in the original manuscript that in subgroup analyses “post-hoc tests show a higher relative risk of meeting the criteria for the risk of kidney dysfunction (RIFLE-R) or kidney injury (RIFLE-I) in the HES group than in the saline group”.

Summarizing the three trials, one showed no relevant differences [13] while two suffered from protocols not reflecting clinical reality, ignored contraindications and maximum recommended daily doses, discarded indications for starches, over-interpreted the results and/or hide important facts in attachments and appendices [11,12]. Importantly, *not one* of them evaluated the initial 6-hour phase shown to be crucial for patient outcome [10]. However, in all trials colloids were given to the majority of patients in this crucial phase, also in the crystalloid groups. In the starch groups, the wrong fluid (the indication suggested crystalloids) in the wrong amount (starches were partly overdosed) at the wrong time (after stabilization) in the wrong patients (many had acute renal failure and most were hemodynamically stabilized) was infused. Therefore, side-effects and complications are no surprise. These trials show that starches should not be infused during sepsis after the initial stabilization phase. With the above-mentioned shortcomings, however, they do not provide evidence for safety issues of starches outside this very special (contra-) indication. Most certainly they do not challenge the knowledge that isooncotic colloids are vital drugs in the perioperative setting. Recent meta-analyses in this collective have shown that non-septic patients receiving 6% HES 130 with a proper indication could have a superior risk/benefit ratio and improved outcome compared to crystalloids [2,15].

## Conclusion: what will the future bring?

In contrast to oral discussions and opinion papers, the EMA has the power to dictate behaviour to clinicians. This is associated with a great responsibility. Therefore, in our understanding such an authority should stand clear from controversial discussions among experts, avoiding extrapolation of data from one to another clinical situation and keeping to facts. As the official recommendation in it’s current form is not based on reliable data it arbitrarily takes an important drug out of the hands of physicians who do exactly what they should do: Pay attention to physiological principles and stick to the objective available data, to the benefit of their patients. We are quite sure that this was not intended. We hope that the European Medicines Agency will use the current revision procedure for re-thinking and getting back from a politically driven to a scientific issue, limiting the use of starches to an indication-centred one.

## Competing interests

The authors have held lectures for and received research grants from Baxter (Unterschleißheim, Germany), B Braun, Melsungen (Melsungen, Germany), Fresenius Kabi (Bad Homburg, Germany), Grifols (Barcelona, Spain) and Serumwerk Bernburg (Bernburg, Germany). MJ is member of the Albumin Advisory Board of Grifols (Barcelona, Spain).

## Authors’ contributions

Both authors designed, wrote and discussed this comment. Both authors read and approved the final manuscript.
